# Therapeutic Effect of α-MSH in Primary Cultured Orbital Fibroblasts Obtained from Patients with Thyroid Eye Disease

**DOI:** 10.3390/ijms222011225

**Published:** 2021-10-18

**Authors:** Pei-Wen Cheng, Pei-Jhen Tsai, Ming-Hong Tai, Youn-Shen Bee

**Affiliations:** 1 Department of Medical Education and Research, Kaohsiung Veterans General Hospital, Kaohsiung 813414, Taiwan; pwcheng@vghks.gov.tw; 2 Department of Ophthalmology, Kaohsiung Veterans General Hospital, Kaohsiung 813414, Taiwan; pjtasi@vghks.gov.tw; 3 Department of Biological Sciences, National Sun Yat-Sen University, Kaohsiung 813414, Taiwan; 4 Institute of Biomedical Sciences, National Sun Yat-Sen University, Kaohsiung 813414, Taiwan; minghongtai@gmail.com; 5 Department of Optometry, Shu-Zen Junior College of Medicine and Management, Kaohsiung 813414, Taiwan

**Keywords:** thyroid eye disease, orbital fibroblasts, proopiomelanocortin (POMC), α-melanocyte stimulating hormone (α-MSH), inflammation

## Abstract

Inflammation, hyaluronan production, and adipogenesis are the main pathological events leading to thyroid eye disease (TED). α-Melanocytemelanocyte-stimulating hormone (α-MSH) is a well-known tridecapeptidetreatment for several inflammatory disorders including sepsis syndrome, acute respiratory distress syndrome, rheumatoid arthritis, and encephalitis. Here, we investigated the effect of α-MSH treatment on TED. The 3-(4,5-Dimethylthiazol-2-yl)-2,5-diphenyltetrazolium bromide (MTT) and Lactate Dehydrogenase (LDH) assays were performed to analyze the effect of α-MSH on cell viability and it’s toxicity. Using primary cultures of orbital fibroblasts from TED patients and non-TED as control, we examined the effects of α-MSH on proinflammatory cytokine production induced by interleukin (IL)-1β, further analyzed by real-time reverse transcription-polymerase chain reaction (qPCR) and western blotting. Immunofluorescence staining assay and qPCR were performed to examine proopiomelanocortin (POMC) expression, the upstream neuropeptide of α-MSH in TED patients and non-TED control. Treatment with non-cytotoxic concentrations of α-MSH resulted in the dose-dependent inhibition of mRNA and protein levels (*p* < 0.05) for IL-1β-induced inflammatory cytokines: IL-6, IL-8, MCP-1, ICAM-1, and COX-2. The expression of POMC mRNA and protein were significantly higher in TED patients compared to non-TED control (*p* < 0.05). Our data show significant inhibitory effects of α-MSH on inflammation, POMC production in orbital fibroblasts. At present, this is the first in vitro preclinical evidence of α-MSH therapeutic effect on TED. These findings indicate that POMC and α-MSH may play a role in the immune regulation of TED and can be a potential therapeutic target.

## 1. Introduction

Thyroid eye disease (TED), also known as thyroid-related ophthalmopathy (TRO), Graves’s ophthalmopathy (GO), is an autoimmune inflammatory disease of the orbit [1,2]. TED presents a wide spectrum of clinical severity ranging from subjective irritation to significant extraocular muscle enlargement, proptosis, ophthalmoplegia, exposure keratitis, and optic neuropathy (ON). The inflammatory process in the orbit affects muscles, connective and adipose tissues at various degrees, which is responsible for the several clinical manifestations of the disease [3]. Enlargement of extraocular muscle bodies together with an increase in orbital connective/fatty tissue within the bony orbits was responsible for most of the orbital complications in patients having moderate to severe TED [4].

Previous research showed that TED is related to the abnormal secretion of inflammatory cytokines [5,6]. These over-expressed inflammatory cytokines promoted the infiltration of thyroid lymphocytes and the activation of B cells, leading to the production of autoimmune antibodies against thyroid antigens during TED development [7]. Under influence of pro-inflammatory cytokines, orbital fibroblasts from TED patients produced excess glycosaminoglycan and inflammatory cytokines such as interleukin IL-6 and IL-8 [8,9]. Furthermore, the expression levels of intercellular adhesion molecule-1 (ICAM-1) and cyclooxygenase (COX)-2 in the orbital connective tissue of TED patients were up-regulated [7,10,11].

Clinical therapy for ocular inflammatory diseases, mainly glucocorticoids, is effective in general, however, this comes with adverse effects. Consideration of alternative treatment targets, such as the melanocortin pathway, can be of value to patients who experience suboptimal response resultingfrom traditional treatment or who have intolerable adverse effects fromtraditional treatments to minimize inflammation [12]. The tridecapeptide α-melanocyte-stimulating hormone (α-MSH) is the precursor for proopiomelanocortin (POMC) and belongs to melanocortin family [13]. The production of α-MSH is widespread and mainly generated by the pituitary, skin tissue, and immune cells [13], it’s anti-inflammatory and immunomodulatory properties have been the focus of study in recent years [14,15]. Pharmacologically, α-MSH is extremely effective in preclinical treatment of local and systemic inflammatory disorders such as sepsis syndrome, acute respiratory distress syndrome, rheumatoid arthritis, inflammatory bowel disease, encephalitis [13,16]. One study demonstrates that α-MSH intravitreal injections exert anti-oxidative and anti-apoptotic effects in retinal vessels and neuroretina of diabetic rats. In addition, α-MSH reduces pro-inflammatory microenvironment in diabetic retinas [17], regulates ocular immune function in the eyes, and also prevents chronic inflammation in the eyes [12,18]. Recent studies have revealed that α-MSH possesses anti-inflammatory effect, thus, we investigated α-MSH anti-inflammatory effect on TED.

Here, we explored the potential regulatory effect of α-MSH in regards to the main pathological events of TED in primary cultures of orbital fibroblasts isolated from TED patients. Understanding potential regulatory effect of TED may provide the fundamental basis for the different clinical manifestations of TED in the future.

## 2. Results

### 2.1. Characterization of Fibroblasts and Endothelial Cells in TED Patients and Normal Control

**Figure 1:**
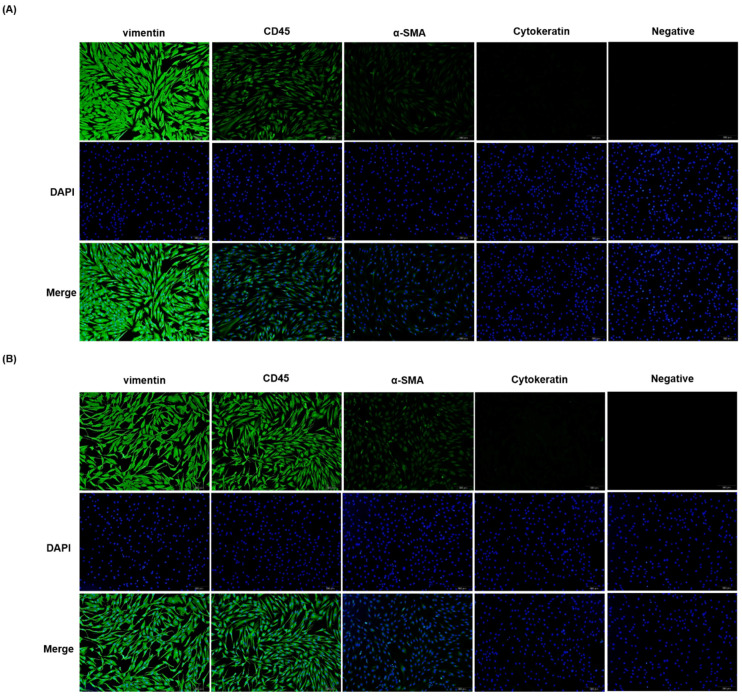
Characterization of orbital fibroblasts isolated from TED patients and control. Orbital fibroblasts were obtained from fat tissue of TED (−) patients (**A**) and TED (+) patients (**B**). Morphology was examined by phase contrast microscopy and the expression patterns for vimentin (VIM), CD45, α-smooth muscle actin (α-SMA), and cytokeratin respectively were examined by immunofluorescence staining in the fibroblast and non-fibroblast. Magnification × 100.

Recently, current concepts in the molecular pathogenesis of thyroid-associated ophthalmopathy indicated that a population of orbital fibroblasts has been putatively traced to bone marrow–derived progenitor cells, known as fibrocytes which expressed CD45, CD34, CXCR4, collagen I, functional TSHR, and thyroglobulin (Tg) [19]. Fibrocytes increase in Graves’ disease (GD), thus we believe that fibrocytes might be linked to the orbit in thyroid-associated ophthalmopathy (TAO).The orbital fat primary culture from normal control and TED patients were flat and elongated in morphology. We examined the expression of fibroblast markers in orbital fibroblasts by immunostaining. As shown in Figure 1, the orbital fibroblasts stained positive for vimentin, CD45, α-SMA, and negative for cytokeratin. The characteristics of primary cell cultured from orbit fat tissues were compatible with fibroblasts. The CD45 and α-SMA signal were higher in TED indicating more fibrocytes and myofibroblasts compared to control. The Figure 1B showed the cell characteristics of TED.

### 2.2. Effect of α-MSH on the Viability of Orbital Fibroblasts

**Figure 2:**
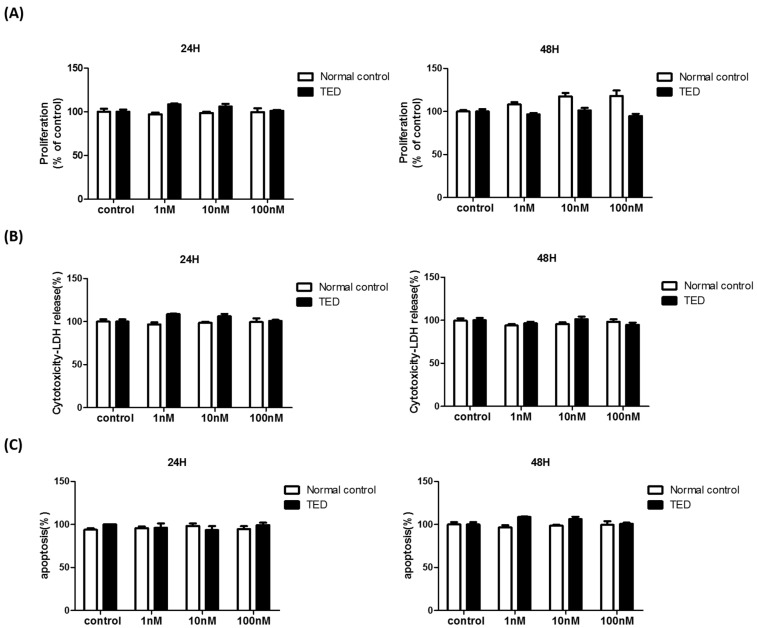
Effect of α-MSH on the viability of primary orbital fibroblasts. Orbital fibroblasts of normal control (non-TED) and TED patients were treated with different concentrations of α-MSH (0–100 nM) for 24 and 48 h. (**A**) MTT assays, used to evaluate viability, were performed in triplicates using cells from different donors. (**B**) LDH assay was performed to testcytotoxicity. (**C**) Annexin V–FITC kit was used to detect phosphatidylserine externalization as an index for apoptosis. The percentage of stained cells with Annexin V was analyzed by flow cytometry. The data was represented in percentage of control (mean ± SEM of six independent experiments, performed in triplicates).

To determine the non-toxic concentrations of α-MSH in normal and TED fibroblasts, MTT assay, LDH assay, and annexin V-fluorescence isothiocyanate (FITC) apoptosis assays were performed. In MTT assay, it was observed that α-MSH (0–100 nM) for 24 and 48 h had no significant effect on cell proliferation in the normal control and TED orbital fibroblasts (Figure 2A). Release of LDH from cells treated with α-MSH (0–100 nM) for 24 and 48 h did not induce cell cytotoxicity in both normal control and TED orbital fibroblasts (Figure 2B). Treatment of α-MSH (1–100 nM) for 24 or 48 h did not induce significant cell apoptosis in the normal control and TED orbital fibroblasts (Figure 2C). The maximal non-cytotoxic concentration of α-MSH was determined to be 100 nM for 24 h in normal and TED orbital fibroblasts, and the concentration of α-MSH 0.1–10 nM was used in the subsequent therapeutic assays.

### 2.3. α-MSH Attenuates the IL-1βInduced Pro-Inflammatory Cytokines Both in Normal and TED Orbital Fibroblasts

**Figure 3:**
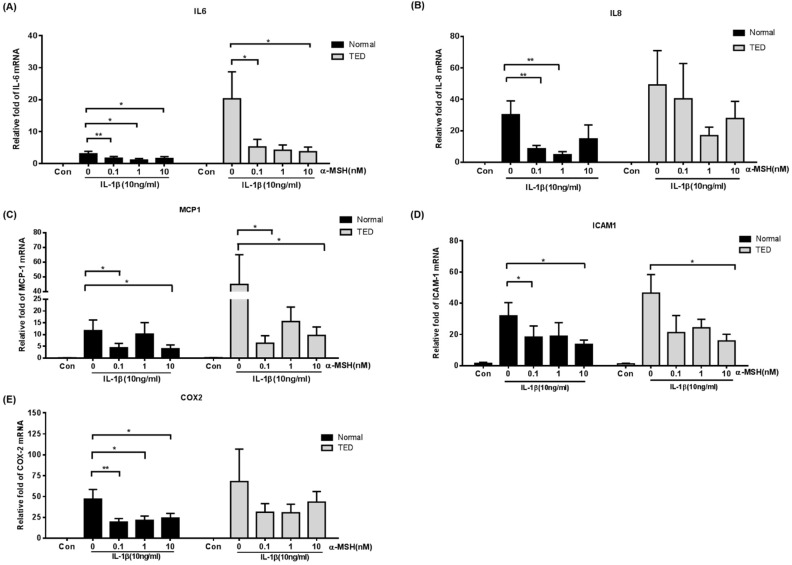
Effect of α-MSH on IL-1β-induced IL-6, IL-8, MCP-1, ICAM-1 and COX-2mRNA expression in normal and TED orbital fibroblasts. Orbital fibroblasts obtained from normal or TED subjects were given 10 ng/mL IL-1β for 16 h with α-MSH (0–10 nM) pretreatment for 6 h. IL-6 (**A**), IL-8 (**B**), MCP-1 (**C**), ICAM-1 (**D**) and COX-2 (**E**) mRNA levels were evaluated using qPCR. Gene transcriptional levels of the cytokines are shown as mean ± SEM fold change in cytokine mRNA levels relative to control without α-MSH treatment. Experiments were performed in triplicates using cells from six different donors. * *p* < 0.05, ** *p* < 0.01 as compared to cells withoutα-MSH.

Previous studies confirmed that interleukin-1 beta (IL-1β) have pleiotropic effects on orbital fibroblasts, stimulating both hyaluronic acid production and adipogenesis to perpetuate orbital inflammation [20,21]. Therefore, we investigated α-MSH effects on IL-1β-upregulated secretion of pro-inflammatory cytokines in normal and TED orbital fibroblasts. We examined different doses of α-MSH (0–10 nM, 6 h and 24 h) stimulation on IL-6, IL-8, MCP-1, ICAM -1, and COX -2 mRNA levels in response to IL-1β (10 ng/mL, 16 h)in both normal and TED orbital fibroblasts by qPCR. Figure 3 shows that α-MSH pretreatment significantly attenuated IL-6, IL-8, MCP-1, ICAM-1, and COX-2 in normal orbital fibroblasts (*p* < 0.05), and attenuated IL-6, MCP-1, and ICAM-1 in TED orbital fibroblasts in a dose-dependent manner at 6 h (*p* < 0.05) (Figure 3A–E). However, administration of α-MSH did not attenuate the levels of IL-6, IL-8, MCP-1, ICAM-1, and COX-2 in both normal and TED orbital fibroblasts at 24 h (Figure S1).

### 2.4. α-MSH Attenuates the IL-1βInduced Expression of COX-2 Proteins Both in Normal and TED Orbital Fibroblasts

**Figure 4:**
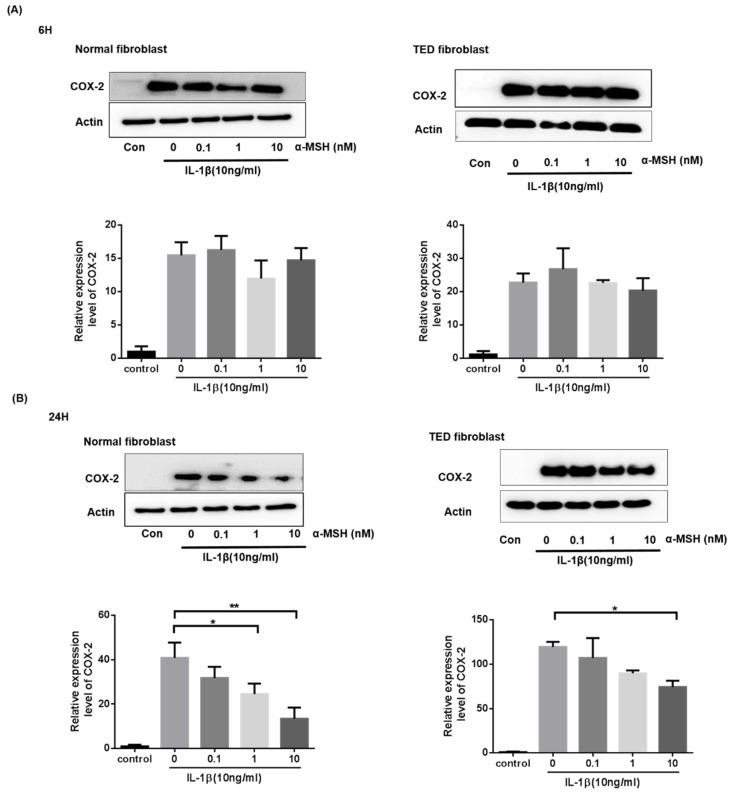
Effect of α-MSH on IL-1β-induced expression of COX-2 proteins in normal and TED orbital fibroblasts.α-MSH suppressed the expression of IL-1β-induced COX-2 in orbital fibroblasts. Orbital fibroblasts cells were stimulated with IL-1β (10 ng/mL), and α-MSH (0–10 nM) for 6 (**A**) and 24 h (**B**). COX-2 expression was assayed by western blot analysis. The graph shows representative data expressed as mean ± standard error for the mean of three independent experiments. * *p* < 0.05, ** *p* < 0.01 compared to cells withoutα-MSH.

Previous studies have shown that cyclooxygenase (COX)-2 in the orbital connective tissue of TED patients were upregulated, and we investigated its expression in the orbital fibroblasts. Orbital fibroblasts were treated with various concentrations of α-MSH (0.1–10 nM) for 6 h and 24 h. As shown in Figure 4, immunoblotting analysis of proteins extracted from the normal and TED orbital fibroblasts demonstrated that a dose-dependent effect of α-MSH suppressed COX-2 protein expression in the normal and TED group at 24 h (*p* < 0.05) (Figure 4).

### 2.5. POMC Expression in Primary Orbital Fibroblasts and Orbital Tissue

**Figure 5:**
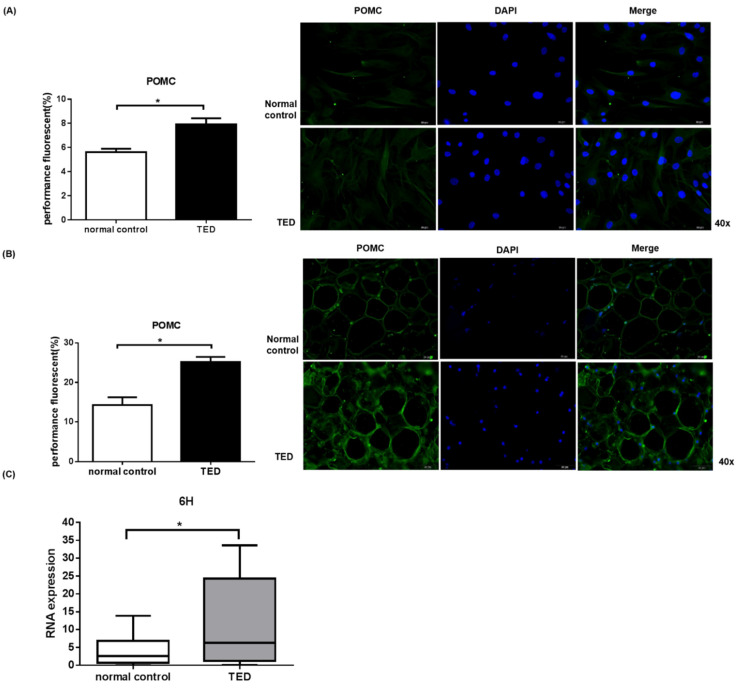
The expression of POMC in orbit fibroblasts and orbit fat tissue from TED and control patients.Immunofluorescence staining assay was performed to examine the expression of POMC levels in primary cultured orbital fibroblasts (**A**) and in orbital fat tissue (**B**). It was found that the expression of POMC in TED was increased compared to the normal control. qPCR assay was performed to examine the POMC mRNA level in orbital fibroblasts. The average SEM multiples of POMC gene levels in TED were higher compared to normal control (**C**). * *p* < 0.05 compared to normal control.

Evidence indicates that α-MSH is a tridecapeptide from the POMC and belongs to the melanocortin family [13]. Production of α-MSH is widespread and mainly generated by the pituitary, skin tissue, and immune cells [13]. The above results show that α-MSH reduced IL-1β-induced inflammatory in both normal and TED orbital fibroblasts. Therefore, we speculated that POMC overproduction may help to regulate TED in vivo. Here, we performed immunofluorescence staining to examine POMC expression in orbital fibroblasts and in orbital fat tissue (Figure 5).The POMC expression in TED orbital fibroblasts and orbital tissues were significantly higher compared to control (*p* < 0.05) (Figure 5A,B). Similarly, the POMC mRNA expression in TED orbital fibroblasts was significantly higher compared to control (*p* < 0.05) (Figure 5C). Surprisingly, POMC expression level was high in the TED patients.

## 3. Discussion

TED is a complex autoimmune disease characterized by chronic inflammation of orbital soft tissue with subsequent tissue remodeling and fibrosis [1,2]. Previous studies have demonstrated that chronic inflammation correlated to the development of TED and the protective effects of anti-inflammatory mediators [22]. In general, several treatment strategies are available according to various disease activity and severity, such as corticosteroid therapy, orbital radiotherapy, selenium administration, and surgical approach [22]. Clinically, corticosteroid therapy has been the major treatment for TED [23,24], whereas management for moderate to severe TED using steroids has not been satisfactory [22]. In addition to corticosteroid therapy, several immunotherapies such as Tocilizumab, Rituximab, and Teprotumumab were also performed [22,25]. Tocilizumab, an IL-6 receptor monoclonal antibody which has been applied in few clinical case studies, has been demonstrated to reduce inflammation, and reduction in Thyroid-Stimulating Immunoglobulin (TSI) was also observed following tocilizumab therapy [22,25]. Nevertheless, tocilizumab did not significantly improve diplopia [26]. Rituximab, the chimeric monoclonal antibody targeting CD20 that causes B cell depletion, might affect autoantibodies binding to the TSH receptor. This Rituximab was previously proposed to be a potential treatment for TED [22,27]. Previous studies pointed out that rituximab has no improvement in the serious consequences of TED (such as exophthalmos and diplopia), and there are the risks for infection, alongside cancer and allergic reactions [28]. Teprotumumab, a human monoclonal IGF-1R antibody formerly approved by FDA for TED in January 2020 [29,30], was shown to attenuate the effects of IGF-1 and thyroid stimulating hormone (TSH) in fibroblasts [22,31]. In a randomized, placebo-controlled, phase 3 multicenter trial, patients with active thyroid eye disease, teprotumumab resulted to improved outcome with respect to proptosis, Clinical Activity Score, diplopia, and quality of life compared to placebo [30]. Even steroids remained the major medication, more immunomodulator agents were used to treat TED [32]. Recently, understanding of the molecular basis of TED hasled to the development of immunomodulatory agents, indicating that there may yet be a viable therapeutic option for patients with active TED [22].

In this study, we investigated the role of α-MSH on the expression of pro-inflammatory cytokines in orbital fibroblast from TED patients. Recently, α-MSH potential anti-inflammatory function both in vitro and in vivo has gained increased attention [33]. An animal study has proven that α-MSH suppressed endotoxin- and pro-inflammatory cytokine-(such as IL-1β and TNF-α) induced systemic inflammatory responses [34]. Apart from this, α-MSH was also effective in relieving ocular anti-inflammatory effect in endotoxin-induced uveitis. Mechanistically, α-MSH is believed to carry out anti-ocular inflammatory effect through inhibition of PGE2, TNF-α, IL-6, and MCP-1 productions, and prevent the expression of COX-2 [35]. In our study, application of α-MSH to primary cultured orbital fibroblasts did not result incell viability change. Pretreatment with α-MSH significantly attenuated the mRNA levels of IL-6, IL-8, MCP-1, ICAM-1, and COX-2 in normal orbital fibroblasts, and α-MSH pretreament significantly reduced IL-6, MCP-1, and ICAM-1 in TED orbital fibroblasts. Nevertheless, α-MSH significantly suppressed IL-1β-stimulated COX-2 protein upregulation in both normal and TED fibroblasts, confirming that α-MSH exhibited significant suppressive effects on IL-1β stimulated inflammation of orbital fibroblasts in TED.

Melanocortin was found to transiently decrease the intraocular pressure (IOP) instead of increase IOP present in the steroid therapy [36]. The anti-inflammatory actions of steroids would suppress the immune system, but α-MSH appear to have multiple pathways for immune modulation of acute inflammation with pro-resolving properties that alter the phenotype of immune cells, allowing them to become prone to being modulated [18]. The POMC gene is mainly expressed in neurons from the hypothalamus and pituitary. It is also found in different nuclei of the central nervous system, various peripheral tissues (such as skin, kidney, and liver) and cells of the immune system [37]. POMC is processed into various neuropeptides including adrenocorticotrophic hormone (ACTH); α-, β-, and γ-melanocyte-stimulating hormone (MSH); and β-endorphin. POMC-derived peptides possess pleiotropic functions, including pigmentation, adrenocortical function, regulation of energy homeostasis, and immunity modulation, in the central and peripheral system [38,39].

POMC mRNA is detected in the peripheral immune cells: lymphocytes and monocytes, suggesting that POMC-derived peptides have a regulatory role in the inflammation of cytokines, interferon or hormone-induced signal transduction activation, and transcriptional signal transduction activators can enhance POMC expression and melanocortin synthesis at sites of infection or inflammation [40]. Anti-inflammatory and immunosuppressive functions of POMC neuropeptides have been previously reported [41]. Stress hormone POMC is the precursor for several anti-inflammatory peptides [42], and a previous study revealed that chronic inflammation of hypothalamic neurons resulted inmetabolic disease which is correlated to leptin receptor-mediated POMC gene expression [43]. In hypothyroid rats, decreased levels of POMC [44,45] have been found, together with decreased corticosterone levels in the plasma [46]. Conversely, triiodothyronine (T3) treatment tends to increase POMC mRNA levels in hyperthyroid rats [44,47]. We found that the POMC gene expression is upregulated in TED patients’orbital fibroblasts and orbit tissue.

Previous studies have demonstrated that chronic inflammation correlated to the development of TED and the protective effects of several anti-inflammatory mediators [22]. Similarly, our data documentedthatthe levels of inflammatory cytokines in TED orbital fibroblasts were increased compared to control group at 6 h and 24 h (Figure S2). TED is a long-term disease wherein the mRNA expressionat 6 h and protein expression were observed at time point of 24 h. This is the limitation of experiment performed since it is performed in vitro, instream of in vivo. Administration of α-MSH did not lead to significant decrease in cytokines at 24 h, but a decreasing tendency was observed (Figure S1). Hence, the anti-inflammatory trend of α-MSH could be documented as valid for TED. POMC gene showed increased expression in TED patients’orbital fibroblasts and orbit tissue (Figure 5). We speculate that chronic, or more inflammation in TED might compromise the α-MSH anti-inflammatory effect on POMC in TED patients. It is our shortcomings that we have not studied the inflammatory cells which will be included in the future study.

At present, this study is the first report on the function of melanocortins, high POMC expression in TED, and surprising α-MSH anti-inflammatory property in the thyroid eye disease. Based on our observation, we speculate that the hypothalamic-pituitary-adrenal axis and the melanocortin signaling pathway together play a critical role for inflammatory regulation of thyroid eye disease.

## 4. Materials and Methods

### 4.1. Cell and Tissue Culture Protocols

Orbital adipose tissue specimens were obtained from chronic TED patients who underwent orbital or eyelid surgery, and adipose tissue was obtained from patients who have undergone orbital or eyelid surgery without dysthyroid history as control group. Collection of these samples was approved by the Ethics Committee of Kaohsiung Veterans General Hospital and studies were conducted in accordance with the ethical standards in the Declaration of Helsinki (VGHKS15-CT2-15 and VGHKS15-CT7-11). Orbital fibroblast primary cultures performed as previously described [9,48]. Orbital fibroblasts were cultured from orbital adipose connective tissues, obtained from six TED patients (two males, four females, and the mean age of 59 years). The normal control tissues were obtained from six patients without dysthyroid history (three males, three females, mean age of 64). Tissue explants were minced and placed directly in plastic culture dishes to allow pre-adipocyte fibroblasts proliferation. Cells were incubated in Dulbecco’s Modified Eagle Medium (DMEM) containing 10% FBS, penicillin (100 U/mL), and gentamycin (20 μg/mL) in a humidified 5% CO_2_ incubator at 37 °C. Cells of passage 1 to 5 were used in this study. Cultured orbital fibroblasts were pretreated with α-MSH before incubation with recombinant human (rh) IL-1β to study the suppressive effect on inflammation.

### 4.2. Characterization of Orbital Fibroblasts

Orbital fibroblasts characteristics were examined using immunofluorescence staining method. For biomarker characterization, vimentin was used for fibroblasts, CD45 for inflammatory cells and fibrocytes, α-SMA formyofibroblasts, and cytokeratin for keratinocytes [49,50,51]. The cells were fixed in 10% neutral buffered formalin, permeabilized by 0.3% Triton X-100 in PBS. Fixed cells were rinsed in PBS, incubated in blocking solution (1% BSA, 0.3% Triton X-100 in PBS) for 30 min, then incubated overnight in primary antibody at 4 °C. The cells were then washed three times in PBS and incubated in the corresponding Alexa-488-conjugated secondary antibody (Invitrogen, Carlsbad, CA, USA) for 1 h at room temperature. Finally, the cells were rinsed twice in PBS, incubated in 4′,6-diamidino-2-phenylindole(DAPI) for 5 min. Fluorescence images of cells were captured by fluorescence microscope. Negative control was processed without primary antibody.

### 4.3. MTT Assays

To evaluate the effect of α-MSH on cell viability, orbital fibroblasts were seeded into 48-well culture plates (1 × 10^4^ cells/well), treated with different concentrations of α-MSH (0–100 nM) for 24 and 48 h. After treatment, cells were washed, incubated in 0.5 mg/mL 3-[4,5-dimethylthiazol-2-yl]-2,5-diphenyl-tetrazolium bromide (MTT) solution for 4 h at 37 °C. The formazan in viable cells were dissolved by 100 µL of dimethyl sulfoxide, measurement determined by reading optical densities in microplate reader (Multiskan FC, Thermo Scientific, Waltham, MA, USA) at an absorption wavelength of 570 nm.

### 4.4. Lactate Dehydrogenase (LDH) Assay

The cytotoxicity of α-MSH in orbital fibroblasts was determined using the LDH leakage assay. To prepare for the LDH assay, orbital fibroblasts were cultured in media containing varying α-MSH concentrations (0–100 nM) for 24, 48 h. The medium was transferred to a 1.5 mL microcentrifuge tube, centrifuged at 12,000× *g* and 4 °C for 15 min to remove cell debris. Then, 100 μL of each sample was added to the substrate solution and the absorbance at 490 nm was measured using microplate reader (Multiskan FC, Thermo Scientific, Waltham, MA, USA). Activity of LDH was obtained by measuring the decrease in nicotinamide adenine dinucleotide absorbance.

### 4.5. Apoptosis Assays

To evaluate the effect of α-MSH on the apoptosis of orbital fibroblasts, annexin V–FITC kit (BD Biosciences, Franklin Lakes, NJ, USA) was used. Cells were washed in PBS, incubated in serum-free DMEM in the presence of α-MSH at 0 to 100 nM for 6 and 24 h. Cells were washed and incubated for 15 min at room temperature in the presence of FITC-annexin V and propidium iodide (PI). In total, 10,000 cells were excited at 488 nm, and emission was measured at 530 and 584 nm to assess FITC and PI fluorescence, respectively.

### 4.6. Extraction of RNA

The total RNA of the tissue was extracted using TRIzol reagent (Invitrogen, Carlsbad, CA, USA) according to the manufacturer’s instruction. Briefly, tissue or cell samples were homogenized in 1 mL TRIzol reagent and mixed with 0.2 mL BCP to extract protein, before RNA was precipitated in 0.5 mL isopropanol. The concentration, purity, and amount of total RNA were determined using Nanodrop 1000 spectrophotometer (Nanodrop Technologues Inc., Wilmington, DE, USA).

### 4.7. Quantitative Real-Time PCR

Total RNA was isolated and reverse transcribed into complementary DNA according to the manufacturer’s instructions (#4368814; Applied Biosystems, Foster City, CA, USA). The resulting cDNA was amplified on an ABIPrism7700 system (Applied Biosystems, Foster City, CA, USA) using the SYBR green universal PCR master mix and recommended PCR conditions to quantitatively assess gene transcript levels in the cell samples. All PCR reactions were performed in triplicates. The primers were as follows: human IL-6, forward 5′-TCAATGAGGAGACTTGCCTG-3′, reverse 5′-GATGAGTTGTCATGTCCGC-3′; human IL-8, forward 5′-TTGGCA GCCTTCCTGATTTC-3′, reverse 5′-AACTTCTCCACAACCCTCTG-3′;Monocyte Chemoattractant Protein-1 (MCP-1), forward 5′-CAAGCAGAAGTGGGTTCAGGAT-3′, reverse 5′-TCTTCGGAGTTTGGGTTTGC-3′; intercellular adhesion molecule (ICAM) -1, forward 5′-GGCCTCAGCACGTACCTCTA-3′, reverse 5′-TGCTCCTTCCTCTTGGCTTA-3′; cyclooxygenase (COX)-2, forward 5′-GTTCCACCCGCAGTACAG-3′, reverse 5′-GGAGCGGGAAGAACTTGC-3′; and glyceraldehyde-3-phosphate dehydrogenase (GAPDH), forward 5′-TGCACCACCAACTGCTTAGC-3′, reverse 5′-GTCCACCACCCTGTTGCTGTA-3′. The GAPDH expression was used for normalization, results are expressed as fold change in the threshold cycle (Ct) value relative to the control using the 2−ΔΔCt method.

### 4.8. Western Blot Assay

Differentiated cells were washed in ice-cold PBS and lysed in RIPA lysis buffer (50 mMTris–HCl pH 7.4, 1% NP-40, 0.25% sodium deoxycholate, 150 mMNaCl, 1 mM PMSF and protease inhibitors). Lysates were centrifuged for 10 min at 12,000× *g* and the cell homogenate fractions stored at −70 °C. Protein concentrations in the supernatant fractions were determined by the Bradford assay. Equal amounts of protein (30 μg) were boiled in sample buffer and resolved by 10% (wt/vol) SDS-PAGE. Proteins were transferred to polyvinylidene fluoride membranes (PVDF) (Immobilon-P membrane; Millipore, Bedford, MA), probed overnight in primary antibodies in Tris-buffered saline containing Tween 20 (TBST), and washed three times in TBST. Immuno-reactive bands were detected using horseradish peroxidase–conjugated secondary antibody, visualized using enhanced chemiluminescence kit (Amersham Pharmacia Biotech, Piscataway, NJ, USA).

### 4.9. Immunofluorescence Assay

In order to determine POMC expression in orbital fibroblasts and orbital fat tissue, immunofluorescence staining was performed. Then orbital fibroblasts were fixed in 10% neutral buffered formalin and permeabilized by 0.3% Triton X-100 in PBS. Fixed tissue or cells were rinsed in PBS, incubated in blocking solution (1% BSA, 0.3% Triton X-100 in PBS) for 30 min, then incubated in POMC antibody solution at 4 °C overnight. The tissue or cells were washed three times in PBS and incubated in Alexa-488-conjugated secondary antibody (Invitrogen, Carlsbad, CA, USA) for 1 h at room temperature. Finally, the tissue or cells were rinsed twice in PBS, counterstained with DAPI for 5 min, images were taken by fluorescence microscope (Olympus, Tokyo, Japan).

### 4.10. Statistical Analysis

Statistical analysis was performed using SPSS version 18.0 (SPSS Inc., Chicago, IL, USA). All data were presented as means ± SD (standard deviation) or SEM (standard error of the mean) of indicated repeats. The differences between groups were analyzed by ANOVA analysis with two-tailed probability and a *p* value of less than 0.05 is considered significant. The results were representations of at least three independent experiments.

## 5. Conclusions

This study shows that α-MSH has anti-inflammatory effects in primary cultured orbital fibroblasts. In addition, we found that endogenous POMC, the upstream neuropeptide of α-MSH, has higher expression level in TED patients. These new findings indicate that endogenous POMC and α-MSH may play a role in the regulation of TED. Therefore, it is worthwhile to explore the anti-inflammatory effects and regulatory pathway for melanocortins for further understanding and breakthrough.

## Data Availability

All data generated or analysed during this study are included in this published article.

## Supplementary Materials

The following are available online at www.mdpi.com/article/10.3390/ijms222011225/s1.
