# Supplementary material for: Therapeutic Effect of α-MSH in Primary Cultured Orbital Fibroblasts Obtained from Patients with Thyroid Eye Disease
Source: Int J Mol Sci. 2021 Oct 18;22(20):11225. doi: 10.3390/ijms222011225 (PMC8537628; doi:10.3390/ijms222011225)
Supplement: Supplementary file 1 [file ijms-22-11225-s001.zip › ijms-1360148-supplementary.pdf]

Supplementary Materials

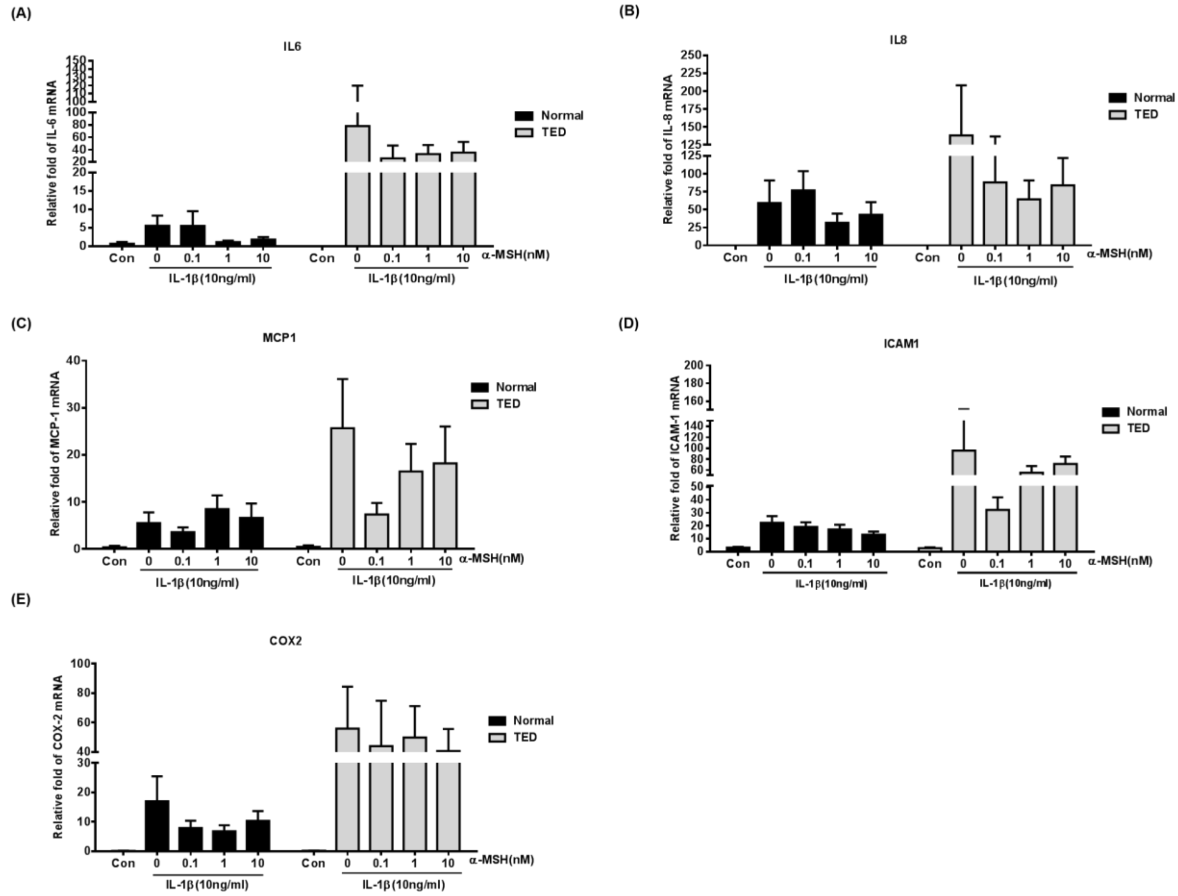

Figure S1. Long-term effect of α-MSH on IL-1β-induced IL-6, IL-8, MCP-1, ICAM-1 and COX-2 mRNA expression in normal and TED orbital fibroblasts. Orbital fibroblasts obtained from normal or TED patients were subjected to 10 ng/mL IL-1β for 16 h, with *α*-MSH (0–10 nM) pretreatment for 24 h. IL-6 (**A**), IL-8 (**B**), MCP-1 (**C**), ICAM-1 (**D**) and COX-2 (**E**) mRNA levels were evaluated using qPCR. Administration of *α*-MSH for 24 h did not attenuate the levels of IL-6, IL-8, MCP-1, ICAM-1, and COX-2 in normal and TED orbital fibroblasts significantly. Gene transcriptional level for cytokines are shown as mean ± SEM fold change in cytokine mRNA levels relative to control without *α*-MSH treatment. Experiments were performed in triplicates with cells from six different donors.

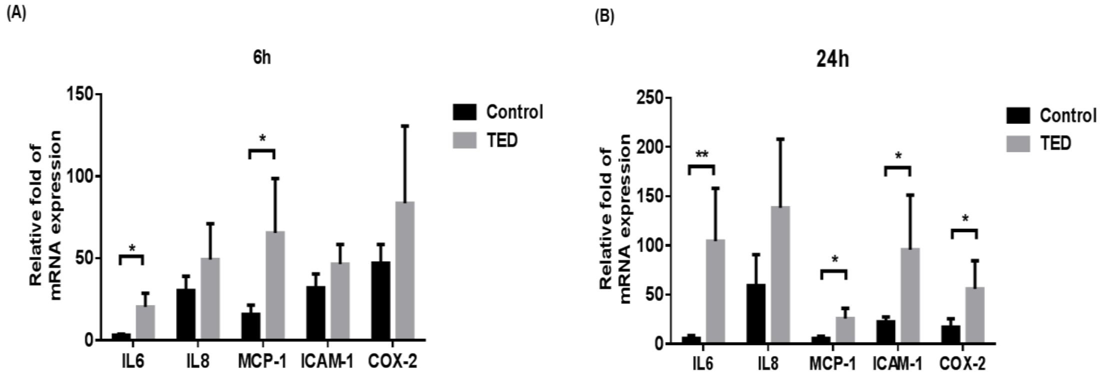

Figure S2. IL-1β-induced IL-6, IL-8, MCP-1, ICAM-1 and COX-2 mRNA expression in normal and TED orbital fibroblasts. Orbital fibroblasts obtained from normal control and TED patients were subjected to10 ng/mL IL-1β for 16 h in 6 h (**A**) and 24 h (**B**) mRNA levels were evaluated using qPCR. Gene transcriptional level for cytokines are shown as mean ± SEM fold change in cytokine mRNA levels relative to control (relative to the control gene GAPDH). Experiments were performed in triplicates with cells from six different donors. * $p < 0.05$, ** $p < 0.01$ as compared with cells in normal control and TED fibroblasts.
